# Dual Origins of Dairy Cattle Farming – Evidence from a Comprehensive Survey of European Y-Chromosomal Variation

**DOI:** 10.1371/journal.pone.0015922

**Published:** 2011-01-06

**Authors:** Ceiridwen J. Edwards, Catarina Ginja, Juha Kantanen, Lucía Pérez-Pardal, Anne Tresset, Frauke Stock, European Cattle Genetic Diversity Consortium, Luis T. Gama, M. Cecilia T. Penedo, Daniel G. Bradley, Johannes A. Lenstra, Isaäc J. Nijman

**Affiliations:** 1 Smurfit Institute of Genetics, Trinity College Dublin, Dublin, Ireland; 2 Research Laboratory for Archaeology, University of Oxford, Oxford, United Kingdom; 3 Veterinary Genetics Laboratory, University of California Davis, Davis, California, United States of America; 4 Departamento de Genética, Melhoramento Animal e Reprodução, Instituto Nacional dos Recursos Biológicos, Fonte Boa, Vale de Santarém, Portugal; 5 Biotechnology and Food Research, MTT Agrifood Research Finland, Jokioinen, Finland; 6 Area de Genética y Reproducción Animal, SERIDA, Gijón, Spain; 7 Archéozoologie, Archéobotanique, Sociétés, Pratiques et Environnements, CNRS Muséum National d'Histoire Naturelle, Paris, France; 8 Faculty of Veterinary Medicine, Utrecht University, Utrecht, The Netherlands; University of Cambridge, United Kingdom

## Abstract

**Background:**

Diversity patterns of livestock species are informative to the history of agriculture and indicate uniqueness of breeds as relevant for conservation. So far, most studies on cattle have focused on mitochondrial and autosomal DNA variation. Previous studies of Y-chromosomal variation, with limited breed panels, identified two *Bos taurus* (taurine) haplogroups (Y1 and Y2; both composed of several haplotypes) and one *Bos indicus* (indicine/zebu) haplogroup (Y3), as well as a strong phylogeographic structuring of paternal lineages.

**Methodology and Principal Findings:**

Haplogroup data were collected for 2087 animals from 138 breeds. For 111 breeds, these were resolved further by genotyping microsatellites *INRA189* (10 alleles) and *BM861* (2 alleles). European cattle carry exclusively taurine haplotypes, with the zebu Y-chromosomes having appreciable frequencies in Southwest Asian populations. Y1 is predominant in northern and north-western Europe, but is also observed in several Iberian breeds, as well as in Southwest Asia. A single Y1 haplotype is predominant in north-central Europe and a single Y2 haplotype in central Europe. In contrast, we found both Y1 and Y2 haplotypes in Britain, the Nordic region and Russia, with the highest Y-chromosomal diversity seen in the Iberian Peninsula.

**Conclusions:**

We propose that the homogeneous Y1 and Y2 regions reflect founder effects associated with the development and expansion of two groups of dairy cattle, the pied or red breeds from the North Sea and Baltic coasts and the spotted, yellow or brown breeds from Switzerland, respectively. The present Y1-Y2 contrast in central Europe coincides with historic, linguistic, religious and cultural boundaries.

## Introduction

The history of human civilisations has left its footprint in the patterns of genetic variation of livestock species across and within continents [1], [2], [3], [4]. Molecular markers, such as mitochondrial DNA (mtDNA) and autosomal polymorphisms, have been particularly useful in investigating the wild species origin of cattle and the subsequent genetic events that shaped the present pattern of genetic diversity. With regards to cattle in Europe, evidence indicates that: (*i*) Balkan cattle act as reservoir of high genetic diversity [5]; (*ii*) there is a marked contrast between north and south Europe [6], [7], [8]; and (*iii*) central European breeds occupy a separate position relative to Mediterranean and northern European cattle [9].

Although the Fertile Crescent is considered the primary centre of taurine cattle domestication, evidence for independent domestication events in other locals is currently debated [10], [11], [12], [13], [14], [15]. Ancestral taurine mtDNA lineages have been identified, and confirmed differences seen between northern and southern European populations of wild cattle (aurochs): the *B. primigenius* haplogroup P was frequent in northern and central Europe [16], while distinct putative auroch matrilines (haplogroups Q and R) were found in modern southern European populations and it appears that these were sporadically introduced into domestic breeds [11], [12]. MtDNA sequences also reveal that indicine cattle originated from a different wild aurochs population, *Bos primigenius namadicus*, in the Indus Valley approximately 8,000 years before present [17], [18].

Differentiation of paternal lineages via analysis of Y-chromosomal variation adds significantly to what can be inferred from mtDNA and autosomal variation [19]. The absence of interchromosomal recombination outside the pseudoautosomal region (PAR) preserves original arrangements of mutational events, and thus male lineages can be traced both within and among populations. Genetic drift is relatively strong due to the effective population sizes of Y-chromosomes being, at most, 25% of the autosomal effective population size [20]. Effective population size is often reduced further by the relatively high variability of male reproductive success. As a result, the Y-chromosome is a sensitive indicator of recent demographic events, such as population bottlenecks, founder effects and population expansions.

In several herd species, males are more mobile than females and compete for reproduction or, in the case of livestock, are selected on the basis of breeding objectives. So while mtDNA variants stay mostly within the herd, Y-chromosomal variants may reflect the origin of sires as influenced by introgression and upgrading. Indeed, in domestic cattle, a marked difference between the distributions of mitochondrial and Y-chromosomal components has been observed [16], [21], [22], [23], [24]. In an initial survey of European breeds, two haplogroups, Y1 and Y2, were found to be dominant in northern and southern Europe respectively [25]. Comparison of cattle Y-chromosome variation over time suggests that the frequency and distribution of these patrilines varied, which could be related with distinct breeding strategies [26]. In European aurochs, Y2 appears to be predominant [27], but so far it is unclear if there was significant introgression from wild bulls into domestic populations.

In the study of human male lineages, the use of Y-specific microsatellites has allowed for refined analyses of the genetic diversity of paternal lineages that can be found within major haplogroups [28], [29]. Similarly, in cattle, microsatellite analysis has identified several Y-haplotypes in Portuguese [30], northern and eastern European [31], western-continental, British and Sub-Saharan Africa [14] breeds, as well as in American Creole [22] breeds. Even though different sets of markers were used in these studies, and each only partially covered the diversity pattern of the paternal lineages, they have confirmed that Y-markers exhibit a strong phylogeographic structure in cattle.

Here we report Y-haplotype data on 128 European, one African and nine Asian breeds, incorporating also data from the previous reports. This comprehensive study confirms a clear north-south contrast that is accentuated by a homogeneity of Y1 haplotypes in north-central Europe and of Y2 haplotypes in and around the Alpine region, both regions that are thought to be the origin of highly productive cattle breeds. This genetic boundary correlates with historic differences between northern and southern European cultures and we consider that this may have implications for the investigation of dairy QTL variation.

## Results

### Cattle haplogroups defined by Y-SNPs

For 238 males from 30 breeds, Y-chromosomal fragments comprising the *ZFY* (1,219 bp and 1,003 bp), *SRY* (2,644 bp) and *DBY* (also known as *DDX3Y*; 406 bp) genes were sequenced [32]. These sequences contained five mutational differences when compared to the zebu Y3 sequences [30], and all European animals carried either the Y1 or Y2 taurine haplogroups. Interestingly, comparison of the Y3 sequence with an *SRY* sequence from an Indian Sahiwal zebu (GenBank accession number AY079145) [33] revealed three additional differences downstream of the open-reading frame, indicating zebu-specific Y-chromosomal SNPs. In combination with a SNP in *UYT19* [14], [22], [25], [31], three cosegregating mutations differentiate the taurine Y1 and Y2 haplogroups (**Table S1**). A composite microsatellite in *DBY* [25], with one major allele in both Y1 and Y2 and only present in the Italian Maremmana, was not used for differentiation of haplotypes.

**Figure 1:**
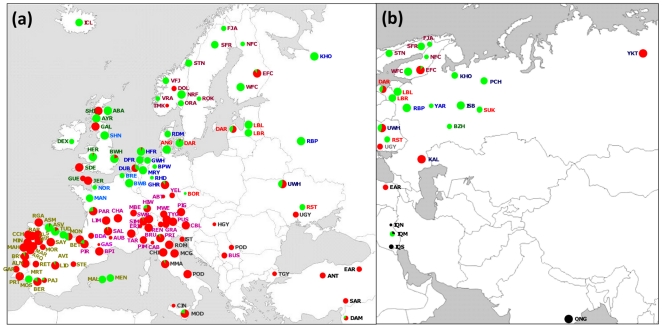
Geographical distribution of Y-haplogroups. (a) Europe, and (b) Eurasia. Green = Y1; red = Y2; black = Y3. Abbreviations of breed names are given in Table 1.

Genotypes of individual SNPs in other animals were combined with Y1-Y2 SNP data and resulted in Y1 or Y2 assignments of 2087 animals from 138 breeds (**Table S1**). The resulting dataset included previously published genotypic information for 1099 individuals from 78 breeds [14], [22], [31]. The Y3 haplogroup was identified on the basis of microsatellite information as described in the next section. The map of **Figure 1** shows the geographical distribution of Y-haplogroups. The three haplogroups described in cattle (Y1, Y2 and Y3) were detected in Southwest Asia, but only Y3 was present in the two Indian breeds analysed, which is in agreement with their zebu morphology. Y1 was predominant in northern Europe and in a number of Iberian breeds. In contrast, Y2 was dominant in most central, Mediterranean and Iberian breeds, but was also found in several British and Nordic breeds. Although only a single African breed was included in this study, both Y1 and Y2 haplogroups were present.

Several of the breeds that do not confirm this northern Y1 – southern Y2 distribution pattern appear to have been subject to recent introgression from breeds with similar coat colour [34], but carrying the other Y-chromosomal haplogroup. Thus, Y2 was introduced in Dutch Belted (DUB) by crossbreeding with belted Galloway bulls. Danish Red bulls probably introduced Y1 into the Sicilian Modicana (MOD). The presence of Y1 in central dairy breeds, such as Simmental (SIM), Pezzata Rossa Italiana (PRI) and Hinderwald (HIW), is probably explained by crossbreeding with Red Holstein sires. Likewise, crossbreeding with Lowland Pied or English cattle probably accounts for the predominance of Y1 in Russian dairy cattle [35]. Y-chromosomes act as a single haplogroup and are, in general, homogeneous at the Y-chromosome variation level. In most cases, the presence of Y1 and Y2 haplotypes in a given breed can be explained by its recent history [14].

### Cattle haplotypes detected through SNPs and microsatellites

The diversity within each haplogroup was further assessed by genotyping *INRA189* [36] and *BM861* [37] Y-specific microsatellite markers. The combination of these data with previously published Y-chromosomal haplotypes [14], [22], [31] yielded haplotypes for a total of 1472 animals from 111 breeds, a subset of the individuals for which SNP information was available. Haplotype composition and absolute frequencies, as well as unbiased estimates of haplotype diversities with the associated standard deviations (SD) are shown for each breed and geographic group in **Table 1**. We found a total of 19 composite Y-haplotypes. The relationship between current haplotype nomenclatures is summarised in **Table S2**. Locus *INRA189* is the most informative marker with 10 alleles, differentiated among five Y1 and nine Y2 haplotypes. For 21 bulls belonging to the Indian and Southwest Asian breed groups, haplogroup Y3 was identified via the *INRA189*-88 bp allele. Microsatellite marker *BM861* defines one additional Y1 and three Y2 additional haplotypes. Haplotype Y1-98-158 is the most frequent within the Y1 haplogroup and is detected in 82% of the animals from this haplogroup across all geographic breed groups with the exception of the Indian and Podolian. Within the Y2 haplogroup, Y2-102-158 and Y2-104-158 haplotypes account for 62% and 29% of the animals respectively. A map showing the distribution of Y-haplotypes is included in **Figure S1**.

**Table 1:** Breed information, including geographical grouping, for the 111 cattle breeds sampled as part of this study, and associated haplotypic data (defined as SNP-*INRA189*-*BM861*) and diversity values.

Geographic grouping	Breed	Code	Country of origin	Number of Y-chromosomes	Haplotype																			haplotype diversity (± SD)	total number of haplotypes
					Y1-94-158	Y1-96-158	Y1-98-158	Y1-98-160	Y1-100-158	Y1-102-158	Y2-80-158	Y2-90-158	Y2-94-158	Y2-96-158	Y2-98-158	Y2-98-160	Y2-100-158	Y2-102-158	Y2-102-160	Y2-104-158	Y2-104-160	Y2-106-158	Y3-88-156		
**India (IND)**	Nellore	NEL	Brazil	12																			12	0.000±0.000	1
	Ongole	ONG	India	4																			4	0.000±0.000	1
			**Total**	**16**																			**16**	**0.000±0.000**	**1**
**Africa (AFR)**	N'Dama	NDM	Guinea	12			2					9					1							0.247±0.184	3
			**Total**	**12**			**2**					**9**					**1**							**0.247±0.184**	**3**
**Southwest Asia (SWA)**	Anatolian Black	ANT	Turkey	5											1		1			2		1		0.300±0.424	4
	Damascus	DAM	Syria	3			1													1		1		0.556±0.416	3
	East Anatolian Red	EAR	Turkey	4														1		2		1		0.278±0.393	3
	Middle Iraqi	IQM	Iraq	4			3																1	0.500±0.000	2
	North Iraqi	IQN	Iraq	1																			1	n/a	1
	South Iraqi	IQS	Iraq	3																			3	0.000±0.000	1
	South Anatolian Red	SAR	Turkey	4																2		2		0.222±0.314	2
			**Total**	**24**			**4**								**1**		**1**	**1**		**7**		**5**	**5**	**0.574±0.192**	**7**
**Podolian (POD)**	Chianina	CHI	Italy	20																20				0.000±0.000	1
	Istrian	IST	Croatia	4																4				0.000±0.000	1
	Marchigiana	MCG	Italy	11																11				0.000±0.000	1
	Maremmana	MMA	Italy	19														19						0.000±0.000	1
	Podolica	PODi	Italy	13														9		4				0.154±0.218	2
	Serbian Podolica	PODs	Serbia	4																4				0.000±0.000	1
	Turkish Grey	TGY	Turkey	3																		3		0.000±0.000	1
	Ukrainian Grey	UGY	Ukraine	5								5												0.000±0.000	1
			**Total**	**79**								**5**						**28**		**43**		**3**		**0.193±0.273**	**4**
**Iberian (IBE)**	Alentejana	ALN	Portugal	34																34				0.000±0.000	1
	Alistana-Sanabresa	ALS	Spain	12														12						0.000±0.000	1
	Arouquesa	ARQ	Portugal	33														25		8				0.126±0.179	2
	Asturiana de los Valles	ASV	Spain	38	26		6											6						0.256±0.202	3
	Asturiana de Montana	ASM	Spain	19	18													1						0.070±0.050	2
	Avilena Negro Iberica	AVI	Spain	7			1											1				5		0.270±0.214	3
	Barrosã	BAR	Portugal	33														4		29				0.073±0.104	2
	Berrenda	BER	Spain	5														3		2				0.200±0.283	2
	Betizu	BEB	Spain	17															17					0.000±0.000	1
	Brava de Lide	BRV	Portugal	26			2											23		1				0.122±0.091	3
	Cachena	CCH	Portugal	25														1		24				0.027±0.038	2
	Garvonesa	GAR	Portugal	6														6						0.000±0.000	1
	Lidia	LID	Spain	66									1				1	64						0.020±0.028	3
	Mallorquina	MAL	Spain	8			8																	0.000±0.000	1
	Marinhoa	MAH	Portugal	17														17						0.000±0.000	1
	Maronesa	MAR	Portugal	23																23				0.000±0.000	1
	Mertolenga	MRT	Portugal	21			7			1					1			2		10				0.378±0.273	5
	Minhota	MIN	Portugal	28														28						0.000±0.000	1
	Mirandesa	MIR	Portugal	23														23						0.000±0.000	1
	Morucha	MOR	Spain	5														5						0.000±0.000	1
	Mostrenca	MOS	Spain	21			21																	0.000±0.000	1
	Pajuna	PAJ	Spain	4			1											2				1		0.444±0.342	3
	Preta	PRT	Portugal	29			1											1		5		22		0.158±0.178	4
	Retinta	RET	Spain	6														4		2				0.178±0.251	2
	Rubia Gallega	RGA	Spain	44														44						0.000±0.000	1
	Sayaguesa	SAY	Spain	8										1				5		2				0.202±0.286	3
	Tudanca	TUD	Spain	10			10																	0.000±0.000	1
			**Total**	**568**	**44**		**57**			**1**			**1**	**1**	**1**		**1**	**277**	**17**	**140**		**28**		**0.335±0.244**	**11**
**Central (CEN)**	Blonde d'Aquitaine	BDA	France	5														3		2				0.200±0.283	2
	Bruna de los Pirineds	BPI	Spain	11														11						0.000±0.000	1
	Busha	BUS	Serbia	5														5						0.000±0.000	1
	Cabannina	CAB	Italy	2														1		1				0.333±0.471	2
	Charolais	CHA	France	31														30	1					0.022±0.030	2
	Limousin	LIM	France	24														24						0.000±0.000	1
	Montbeliard	MBE	France	6														6						0.000±0.000	1
	Parthenaise	PAR	France	15			4											11						0.279±0.198	2
	Piemontese	PIM	Italy	13													2	11						0.094±0.133	2
	Pinzgaur	PGZ	Austria	9														9						0.000±0.000	1
	Pirenaica	PIR	Spain	10														8		2				0.119±0.168	2
	Pustertaler	PUS	Italy	13														13						0.000±0.000	1
	Salers	SAL	France	20			1											19						0.067±0.047	2
	Simmental	SIM	Switzerland	15														15						0.000±0.000	1
	Swiss Brown	SWB	Switzerland	14														14						0.000±0.000	1
	Tarentaise	TAR	France	18														18						0.000±0.000	1
	Tyrolean Grey	TYG	Italy	19													2	17						0.066±0.094	2
			**Total**	**230**			**5**										**4**	**215**	**1**	**5**				**0.056±0.045**	**5**
**British (BRT)**	Aberdeen Angus	ABA	Scotland	27			20	7																0.133±0.188	2
	Ayrshire	AYR	Scotland	16			16																	0.000±0.000	1
	British White	BWH	England	21			16	1										1		3				0.252±0.111	4
	Dexter	DEX	Ireland	4			4																	0.000±0.000	1
	Galloway	GAL	Scotland	9														9						0.000±0.000	1
	Hereford	HER	England	21					20									1						0.063±0.045	2
	Highland	HIG	Scotland	10														10						0.000±0.000	1
	Jersey	JER	Jersey	19																19				0.000±0.000	1
			**Total**	**127**			**56**	**8**	**20**									**21**		**22**				**0.413±0.226**	**5**
**Nordic (NOR)**	Blacksided Troender	STN	Norway	7			7																	0.000±0.000	1
	Doela	DOL	Norway	4							4													0.000±0.000	1
	Eastern Finncattle	EFC	Finland	9			1													8				0.148±0.105	2
	Eastern Red Polled	ORA	Norway	5			5																	0.000±0.000	1
	Fjallnara	FNR	Sweden	3			3																	0.000±0.000	1
	Icelandic	ICL	Iceland	8			8																	0.000±0.000	1
	Northern Finncattle	NFC	Finland	3			3																	0.000±0.000	1
	Norwegian (commercial, hybrid)	NRF	Norway	12			12																	0.000±0.000	1
	Swedish Mountain	SFR	Sweden	8			8																	0.000±0.000	1
	Swedish Red Polled	ROK	Sweden	3			3																	0.000±0.000	1
	Telemark	TEL	Norway	2							2													0.000±0.000	1
	Western Finncattle	WFC	Finland	9			9																	0.000±0.000	1
	Western Fjord	VFJ	Norway	6			3		3															0.200±0.283	2
	Western Red Polled	VRA	Norway	3			2		1															0.222±0.314	2
			**Total**	**82**			**64**		**4**		**6**									**8**				**0.222±0.161**	**4**
**Baltic Red (BHR)**	Angler	ANG	Germany	10			10																	0.000±0.000	1
	Danish Red	RDM	Denmark	19			18											1						0.070±0.050	2
	Latvian Blue (native)	LBL	Latvia	9			9																	0.000±0.000	1
	Latvian Brown (commercial)	LBR	Latvia	8			8																	0.000±0.000	1
	Latvian Danish Red	DAR	Latvia	7			3											4						0.381±0.269	2
	Suksunskaya	SUK	Russia	5			4											1						0.267±0.189	2
	Ukrainian Red Steppe	RST	Ukraine	5		1	4																	0.133±0.189	2
			**Total**	**63**		**1**	**56**											**6**						**0.126±0.090**	**3**
**North-West (NWE)**	Belgian Blue	BWB	France	21		19	2																	0.060±0.085	2
	Belgian Red	BRE	Belgium	4			4																	0.000±0.000	1
	Normand	NOR	France	46			46																	0.000±0.000	1
	Shorthorn	SHN	Belgium	19			19																	0.000±0.000	1
			**Total**	**90**		**19**	**71**																	**0.112±0.159**	**2**
**Lowland Pied (LLP)**	Dutch Belted	DUB	Netherlands	8			3											5						0.357±0.253	2
	Friesian-Dutch	FRH	Netherlands	8			8																	0.000±0.000	1
	German Original Black Pied-West	BPW	Germany	3			3																	0.000±0.000	1
	Groningen Whitehead	GWH	Netherlands	6			6																	0.000±0.000	1
	Holstein Friesian	HFR	Netherlands	65			64									1								0.021±0.015	2
	Jutland (old native)	SJM	Denmark	6			6																	0.000±0.000	1
	Meuse-Rhine-Yssel	MRY	Netherlands	9			9																	0.000±0.000	1
	Red Holstein dual type	RH2	Netherlands	1			1																	n/a	1
			**Total**	**106**			**100**									**1**		**5**						**0.072±0.039**	**3**
**Eastern (EAS)**	Bestuzhev	BZH	Russia	4			4																	0.000±0.000	1
	Istobenskaya	ISB	Russia	9			9																	0.000±0.000	1
	Kalmyk	KAL	Russia	12																12				0.000±0.000	1
	Kholomogorskaya	KHO	Russia	6			6																	0.000±0.000	1
	Pechorskaya	PCH	Russia	7			7																	0.000±0.000	1
	Ukrainian Whiteheaded	UWH	Ukraine	11			4		1									6						0.388±0.276	3
	Yakutian cattle	YKT	Siberia	23																22	1			0.029±0.041	2
	Yaroslavskaya	YAR	Russia	3			3																	0.000±0.000	1
			**Total**	**75**			**33**		**1**									**6**		**34**	**1**			**0.373±0.247**	**5**
**Overall**	**111 breeds**			**1472**	**44**	**20**	**448**	**8**	**25**	**1**	**6**	**14**	**1**	**1**	**2**	**1**	**7**	**559**	**18**	**259**	**1**	**36**	**21**	**0.422±0.269**	

Y-chromosomal diversity within breeds is low (mean of 0.42±0.3) with fixed haplotypes in 65 out of 111 breeds (approximately 59%). Interestingly, the Southwest Asian breed group is the most genetically diverse, with a total of seven haplotypes detected in a limited sample of 24 animals, and an unbiased expected haplotype diversity of 0.57±0.4 (**Table 2**). The Iberian and British groups have intermediate variability, with 11 and five haplotypes observed and diversities of 0.34±0.2 and 0.41±0.3 respectively.

**Table 2:** Haplotypic data for the breeds typed for the Y1-Y2-Y3 SNPs and the two microsatellite loci, *INRA189* and *BM861*.

Geographic grouping	SNPs		Mean number of samples / breed	Microsatellites		Mean number of samples / breed	Total number of haplotypes	Haplotype diversity (± SD)
	Breeds	Samples		Breeds	Samples			
India (IND)	2	16	8.0	2	16	8.0	1	0.000±0.0
Africa (AFR)	1	14	n/a	1	12	n/a	3	0.247±0.2
Southwest Asia (SWA)	7	25	3.6	7	24	3.4	7	0.574±0.4
Podolian (POD)	12	184	15.3	8	79	9.9	4	0.193±0.2
Iberian (IBE)	31	651	21.0	27	568	21.0	11	0.335±0.2
Central (CEN)	30	453	15.1	17	230	13.5	5	0.056±0.1
British (BRT)	10	164	16.4	8	127	15.9	5	0.413±0.3
Nordic (NOR)	14	95	6.8	14	82	5.9	4	0.222±0.2
Baltic Red (BHR)	9	77	8.6	7	63	9.0	3	0.126±0.1
North-West (NWE)	5	126	25.2	4	90	22.5	2	0.112±0.1
Lowland Pied (LLP)	9	208	23.1	8	106	13.3	3	0.072±0.1
Eastern (EAS)	8	74	9.3	8	75	9.4	5	0.373±0.3
**Overall**	**138**	**2087**	**15.1**	**111**	**1472**	**13.3**	**19**	**0.422±0.3**

### Phylogeography of Y-haplotypes

**Figure 2:**
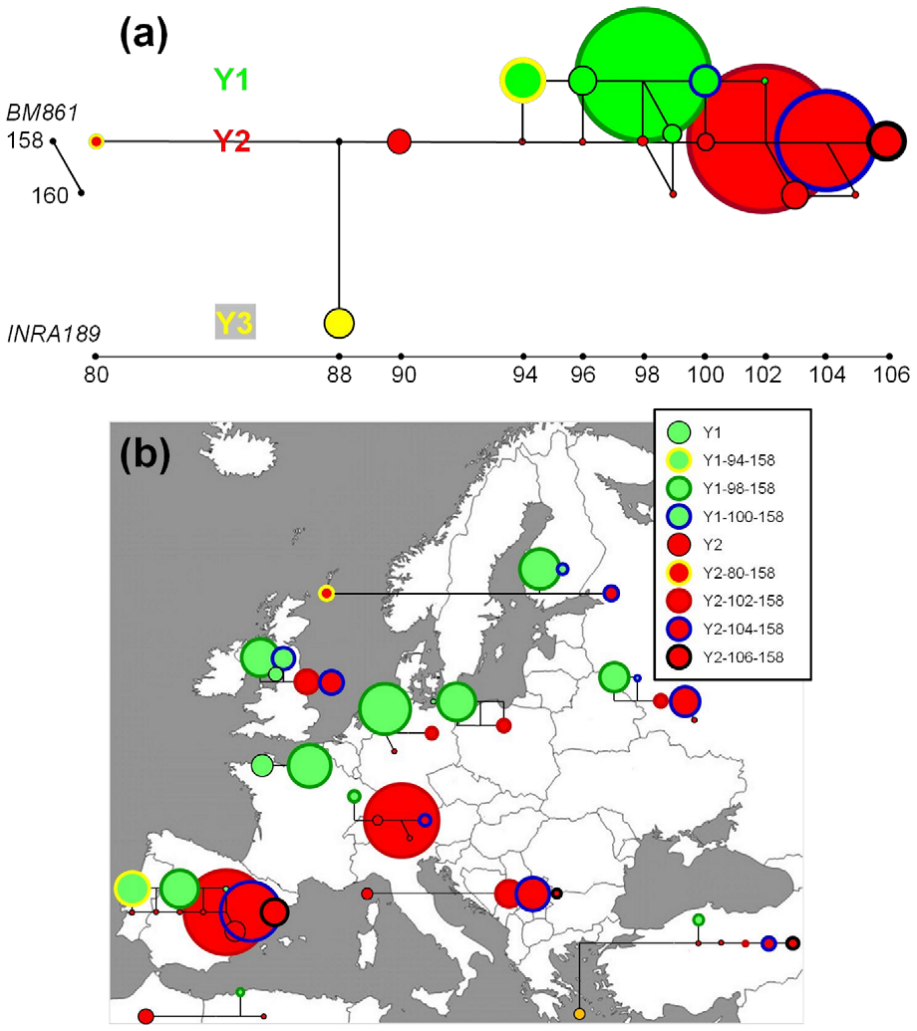
Median-joining networks of Y-chromosome haplotypes using SNPs and two microsatellite markers. The size of each circle represents the number of chromosomes present in each haplotype. (**a**) Haplotype relationships for the complete dataset. (**b**) Regional networks defined for 11 geographic breed groups (India excluded). Haplotypes are indicated with the colouring scheme shown in (**b**).

The phylogenetic relationship among Y-haplotypes was investigated after grouping the breeds into 12 regions on the basis of geography and/or phenotype [34], [38]. These breed groups are in agreement with major clusters as defined by autosomal microsatellite (unpublished results; see also **Figure S2**) and SNP data [7]. Haplotype relationships are depicted in **Figure 2** by a median-joining (MJ) network obtained for the complete dataset, as well as by regional MJ networks defined for each of the 12 geographic breed groups. The MJ networks clearly differentiate the indicine Y3 patriline from the taurine Y1 and Y2 haplogroups. The regional networks depict the Y-chromosome diversity found in each region and the relationship among the observed haplotypes defined by the two microsatellites.

AMOVA results are presented in **Table S3** and show a significant (*P*<0.0001) effect of geographical breed grouping, which accounted for 31% of the total variability. Approximately 51% (*P*<0.0001) of the Y-chromosome genetic variation was found among breeds within groups and 18% (*P*<0.0001) within breeds. This was confirmed by the generally high Y-chromosomal F*st* values (**Table S4**) of all pairwise breed clusters, which were low mainly for the comparison of the dairy north-western European breed clusters.

In **Figure S2**, Y-chromosomal haplotypes have been indicated in a NeighborNet phylogenetic network of Reynolds' distances based on 30 autosomal microsatellite data. This shows a clear consistency between Y-chromosomal variation and the breed relationships in the autosomal-based phylogeny, with only the British Y2 and Iberian Y1 cattle as major exceptions [as in 14], [15].

## Discussion

### Differentiation of paternal lineages in European cattle

We analysed Y-chromosomal haplotypes in a comprehensive sampling of European cattle. This allowed a differentiation of two SNP-based taurine haplogroups, which were resolved into 18 haplotypes using genotypes from two microsatellite markers. Locus *INRA189* appeared to be, by far, the most informative and, in combination with the other marker and SNPs, showed that paternal lineages strongly depend on geographic origin. A further differentiation was demonstrated by using other microsatellites [14], [22], [31]. For instance, *BYM1* alleles defined a Y1-98-158 variant in Nordic cattle and a Y2-102-158 variant in Spain [14], while *DYZ1* detected a different Y2-102-158 variant in Southwest Asian breeds [31].

### Distribution of zebu and taurine haplotypes

Previous genetic studies have demonstrated a separation of the mitochondrial [39], [40], [41], Y-chromosomal [25], [42], [43] and nuclear [21] DNA from taurine and zebu cattle, which supports independent domestications in the Fertile Crescent and Indus valley respectively [44]. In a diverse panel of 2009 European cattle, we exclusively found taurine Y-chromosomal haplotypes. This implies that the zebu alleles found for autosomal markers in the Podolian [6], [45], [46], [47], Iberian [6], [48], [49] and Ukrainian Whitehead [31] breeds came to Europe indirectly, presumably via Anatolia, North Africa or north of the Black Sea. In addition, the presence of zebu Y-chromosomes in Southwest Asia indicate that zebu introgression only took place after the expansion of domestic taurine cattle from Southwest Asia towards Europe [16].

### Taurine haplogroups

The divergence of the two haplogroups Y1 and the Y2 without intermediate haplotypes suggests that domestication combined paternal lineages originate from two diverged populations. Remarkably, both haplogroups were found in Southwest Asia. In Europe, the Y1 and Y2 haplogroups found in extant cattle exhibit a clear geographic structure, with Y1 restricted to northern European and Iberian breeds. Initially, this was explained by local aurochs introgression, supposed to carry the Y1 lineage [25]; however, this does not account for the presence of Y1 in Southwest Asia. In addition, a subsequent study [27] exclusively found Y2 haplogroups in European aurochs. We note that this does not exclude that the Y2 lineage was partially contributed by local aurochs, nor that conditions of Neolithic farming may have created opportunities for male introgression from wild animals. However, several analyses of mtDNA have clearly demonstrated that domestic maternal lineages originate from Southwest Asia, with only sporadic female aurochs introgression [11], [12], [16], [50], [51].

An obvious possibility is that the current Y-chromosomal haplogroup distribution reflects Neolithic immigration routes. According to archaeological evidence, the dispersal of agriculture in Europe started in Greece around 7,000 BC, moved to southern Italy *circa*. 6,000 BC, and then along a southern route into the western Mediterranean between 5,600 and 5,400 BC, reaching Portugal around 5,300 BC. Migration along the continental route into Poland and Germany occurred between 5,500 and 5,300 BC, reaching north-western France around 5,000 BC; southern Scandinavia, the British Isles and Ireland were reached *circa*. or after 4,000 BC [52], [53], [54]. It is generally accepted that agriculture spread via these two routes: the *Mediterranean* route and the *Danubian* (or *continental*) route. Although Y1 has a clear presence in Iberian cattle, Y2 paternal lineages dominate in the present Mediterranean area. A founder effect in Danubian immigrants could have caused the dominance of Y1 in northern Europe. In this scenario, the presence of Y1 in Iberia would have resulted from movements along the Atlantic seaboard, as documented by the Neolithic archaeological record [55], and the presence of two haplogroups in Britain may indicate a convergence of immigrants of both routes.

Alternatively, colonisation of Britain may have predated the expansion of Y1, which could have arrived in Britain later via the documented import of Dutch sires in the 18^th^ century. This would be in line with analysis of skeletal remains excavated in Sweden, showing that Y1 bulls replaced Y2 bulls during or after the late Middle Ages [26], although Y1 cattle were taken to Iceland by the Vikings *c*. 1,000 AD. However, a Y1 founder effect, long after the introduction of domestic cattle in northern Europe, is the most consistent with the haplotype diversity pattern.

Y1 samples identified in Africa are more likely to be the result of a recent introgression of European cattle rather than the expansion of a genetically heterogeneous sire population domesticated in the Fertile Crescent. Y-specific microsatellite data confirm the existence of a Y2 haplotypic subfamily in African cattle restricted to the African continent [14], [15].

### Distribution of haplotype diversity

The comprehensive coverage of our study permits a comparison of the diversity of paternal lineages in different regions. Even in a limited sampling, Southwest Asian cattle contain seven out of the 19 taurine haplotypes identified in this study. This supports the theory that the Fertile Crescent was a major centre of cattle domestication and that European cattle are a subset of an initially diverse Southwest Asian domestic population. The high diversity in Spain and Portugal is probably explained by the isolated position of the Iberian Peninsula, by which much of the original diversity has been conserved. However, the increased diversity may also have been influenced by African introgression, which is consistent with mitochondrial and autosomal information [6], [10], [22], [56], [57]. The diversity in Italian cattle, including the Tuscan breeds with their unusual mtDNA diversity [58], is less than that exhibited in Iberian breeds, with two major Y2 haplotypes. As noted above, the presence of both Y1 and Y2 haplotypes in Britain and the Nordic region may reflect multiple arrivals of cattle, which probably also explains the diversity in Russian cattle.

The diversity in the Mediterranean countries, and in the northern and eastern periphery of Europe, is in striking contrast, with near homogeneity of paternal lineages in north-central and central Europe. The black-pied, red-pied and solid red dairy cattle originating from the Dutch, German and Danish lowlands almost exclusively carry the Y1-98-158 haplotype. We propose that this reflects a founder effect associated with the prehistoric development of dairy farming [59]. Dairy farming by Germanic people in the Roman period has been well documented [Strabo AD23, as translated in 60], while, in the Mediterranean area, cattle were mainly used for draught power and meat consumption [61]. This is consistent with the origin of human lactase tolerance in central Europe [61]. In the 18^th^ century, Rinderpest epidemics led to a replacement of Dutch cattle, with their various coat colours, by black-pied cattle from the Holstein region [34], [62]. These became the ancestors of the highly productive Dutch black-pied cattle, which were exported to several countries. Similarly, the related dairy Red Angler and Danish Red spread to the Baltic countries, Russia and Germany [34], [63].

A different haplotype, Y2-102-158 is predominant in southern France, southern Germany and the Alpine region (**Figure 2**). Many of the present Alpine short-headed cattle are derived from, or related to, the mixed-purpose Simmental and Swiss Brown cattle. It is not known when these types of cattle were developed, although archaeological findings indicate an ancient origin of milk consumption in Switzerland [64] and dairy farming in the Roman period has been documented [Pliny the Elder AD77, as translated in 65]. An increase in size of Swiss cattle during the Roman period further suggests importation of Italian cattle [64]. The predominance of a single Y2 haplotype may indicate again a founder effect. Just as the northern-central European dairy breeds, the Alpine breeds also spread to the surrounding regions. Simmentals were exported to several countries and also used for upgrading of local breeds [34]. Swiss Brown has been crossed with German, Italian and Spanish brown mountain breeds, as well as in the Asturian Valley [66]. We propose that historic or recent spreading of popular breeds acts on genetic diversity patterns and, by the selection of proven sires, has a homogenising effect on paternal lineages.

However, southern French beef breeds also carry exclusively the Y2-102-158 haplotype. A close relationship of southern French and Alpine breeds is indicated by autosomal AFLP profiles [9], SNPs [7] and microsatellite genotypes (unpublished results; **Figure S2**). One explanation might be that contacts of Transalpine Gallic people facilitated gene flow, for instance, during a repopulation of Gallia after the devastating Gallic wars. More recently, the Great Famine, from 1315 to 1317, north and west of the Alps may very well have decimated the French cattle and led to the introduction of Alpine cattle. After the Middle Ages, when the size of cattle started to increase, these would have developed into the present beef types.

The divergence of paternal lineages in north-central and central groups of dairy cattle is fully in agreement with their separate positions as indicated by autosomal markers (**Figure S1**; [7], [9]). This may constitute evidence for independent developments of dairy cattle in different European regions. The Dutch cattle has been crossed with British breeds [34], but not in the Jersey Island dairy cattle, which obviously developed its high milk production separately. An important implication is that in different regions breeding for milk production selected other gene variants, and that crossbreeding of the different categories of dairy cattle is expected to generate potentially useful allelic combinations.

Apparently, the expansion of the dairy breeds have created, or largely maintained, a sharp genetic contrast of northern and southern Europe, which divides both France and Germany. It may be hypothesised that the northern landscapes, with large flat meadows, are suitable for large-scale farming with specialised dairy cattle (*Niederungsvieh*, lowland cattle), whilst the mixed-purpose or beef cattle (*Höhenvieh*, highland cattle) are better suited to the smaller farms and hilly regions of the south. However, it is also remarkable that in both France and Germany the bovine genetic boundary coincides with historic linguistic and cultural boundaries. In France, the Frankish invasion in the north created the difference between the northern *langue d'oïl* and the southern *langue d'oc*. The German language is still divided into the southern *Hochdeutsch* and northern *Niederdeutsch* dialects, which also correlates with the distribution of the Catholic and Protestant religions. On a larger scale, it is tempting to speculate that the difference between two types of European cattle reflects, and has even reinforced, the traditional and still visible contrast of Roman and Germanic Europe.

We conclude that analysis of paternal lineages contributes to a reconstruction of the history of cattle husbandry. A differentiation of more haplotypes [14], [15], [22], [31], and especially the analysis of historic DNA, may answer the question of the European aurochs contribution, as well as indicating a time depth for the successive genetic events that have created the present pattern of genetic diversity.

## Materials and Methods

### Sample collection

Details on sample collection and DNA isolation can be found in previous publications from the participating laboratories [14], [22], [31], [67]. Breeds, geographic origins and sample sizes are shown in **Table S1**. This dataset comprises a total of 138 breeds and 2087 individuals. Apart from the 16 zebu (*Bos indicus*) samples from India, all animals were *Bos taurus*. Based on breed histories and geographical origin, a total of 12 geographic breed groups were defined as follows: India (2 breeds), Africa (1), Southwest Asia (7), Podolian (12), Iberian (31), Central (30), British (10), Nordic (14), Baltic Red (9), North-West (5), Lowland Pied (9) and Eastern (8).

### Sequencing and genotyping

Segments of the *ZFY*, *SRY* and *DBY* genes were PCR-amplified and sequenced as previously described [32]. As indicated in **Table S1**, individual SNPs were typed by pyrosequencing [25], [30], sequencing [14] or by custom service via the Taqman (Van Haeringen Laboratory, Wageningen, Netherlands) or KASPar (K-Bioscience, Hoddesdon, UK) procedures. Several samples were analysed by more than one method.

The Y-chromosomal microsatellites *BM861* [37], *INRA189* [36], *INRA124* and *INRA126* [68] were genotyped as described in previous publications [14], [30], [31], [67]. Results from *INRA124* and *INRA126* were discounted due to these markers showing amplification in female samples [69]. Allele sizes were standardised via known fixed alleles from several samples or breeds analysed by the different laboratories (Dublin, Jokioinen, Davis and Gijon). Y-haplotypes were defined for each individual by combining data from both SNPs and microsatellite loci (SNP-*INRA189*-*BM861*).

Genotyping of the FAO-recommended autosomal microsatellites has been described previously [5], [35], [70].

### Statistical analyses

Variability at the DNA level was assessed in each breed and within each geographic group by estimating the unbiased expected genetic diversity [71], and by an analysis of molecular variance (AMOVA), using ARLEQUIN version 2.0 [72]. Phylogenetic relationships among haplotypes were analysed by constructing median-joining (MJ) networks for each region using the algorithm of Bandelt *et al.* [73], as implemented in the NETWORK (Version 4.156; www.fluxus-engineering.com).

## Supporting Information

Figure S1 **Map showing distribution of SNP Y-haplotypes in: (a) Europe, and (b) Eurasia.** Haplotypes are indicated with the colouring scheme shown in **(b)**. Abbreviations of breed names are given in Table 1.
(PDF)

Figure S2 **NeighborNet graphs of Reynolds' distances, based on 30 autosomal microsatellites.** Haplotypes are indicated with the same colouring scheme as for: (**a**) maps in **Figure S1**, and (**b**) maps in **Figure 1**. Abbreviations of breed names are given in Table 1.
(PDF)

Table S1 (DOC)

Table S2 (DOC)

Table S3 (DOC)

Table S4 (DOC)
