# Supplementary figures and images for: Dual Origins of Dairy Cattle Farming – Evidence from a Comprehensive Survey of European Y-Chromosomal Variation
Source: PLoS One. 2011 Jan 6;6(1):e15922. doi: 10.1371/journal.pone.0015922 (PMC3016991; doi:10.1371/journal.pone.0015922)

(a)

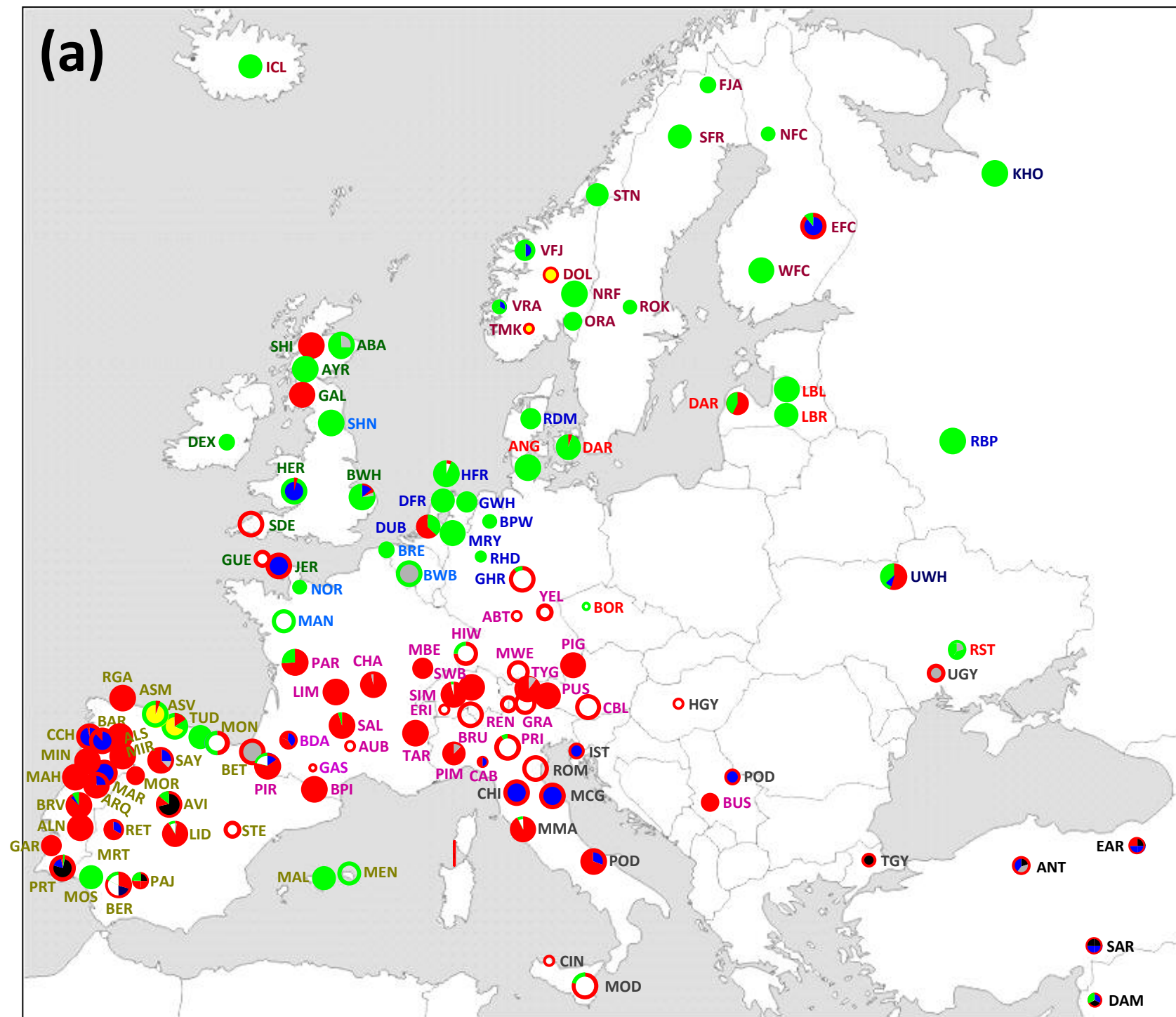

(b)

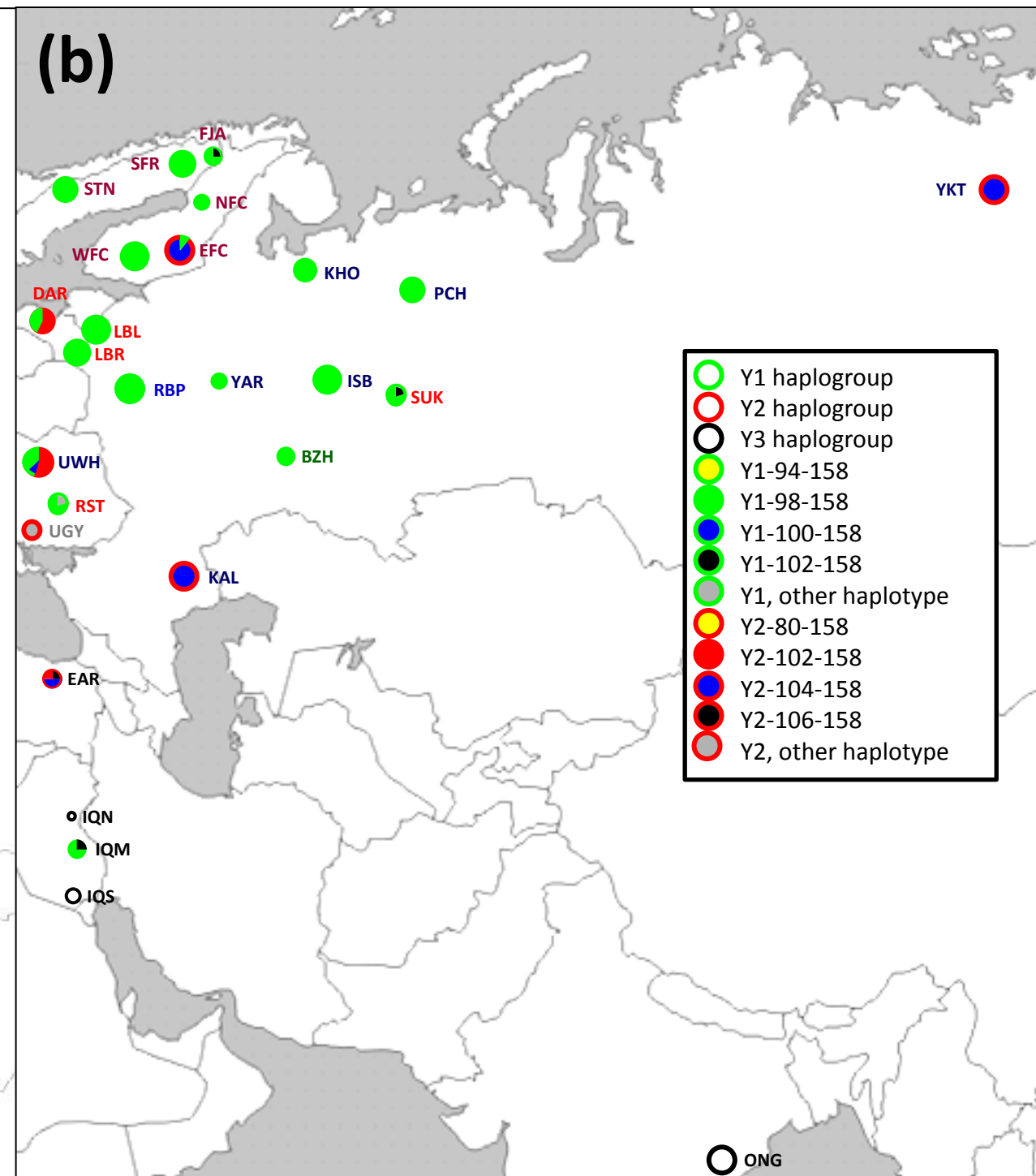

Supplement: Figure S1 — Map showing distribution of SNP Y-haplotypes in: (a) Europe, and (b) Eurasia. Haplotypes are indicated with the colouring scheme shown in (b). Abbreviations of breed names are given in Table 1. (PDF) [file pone.0015922.s001.pdf]

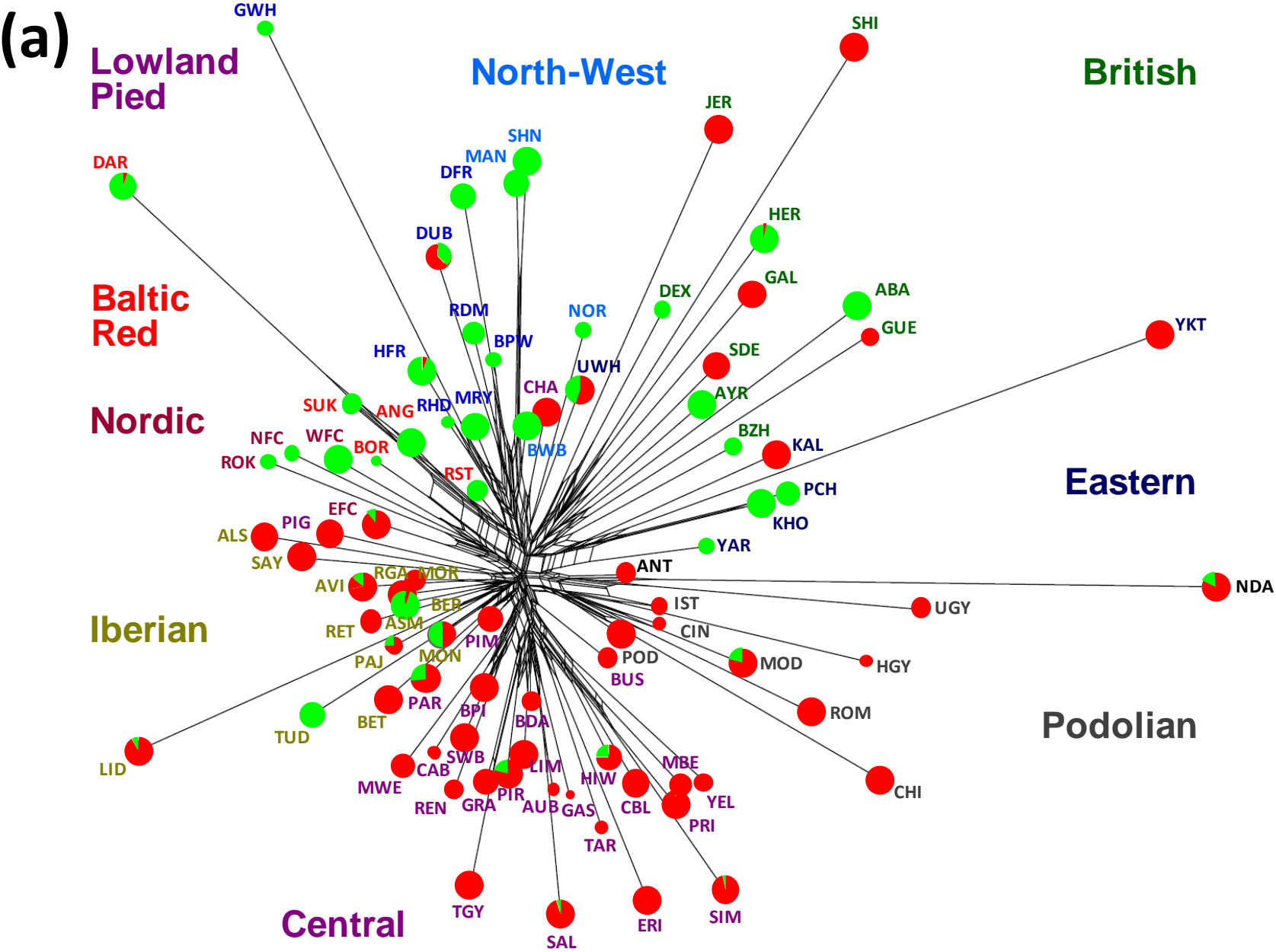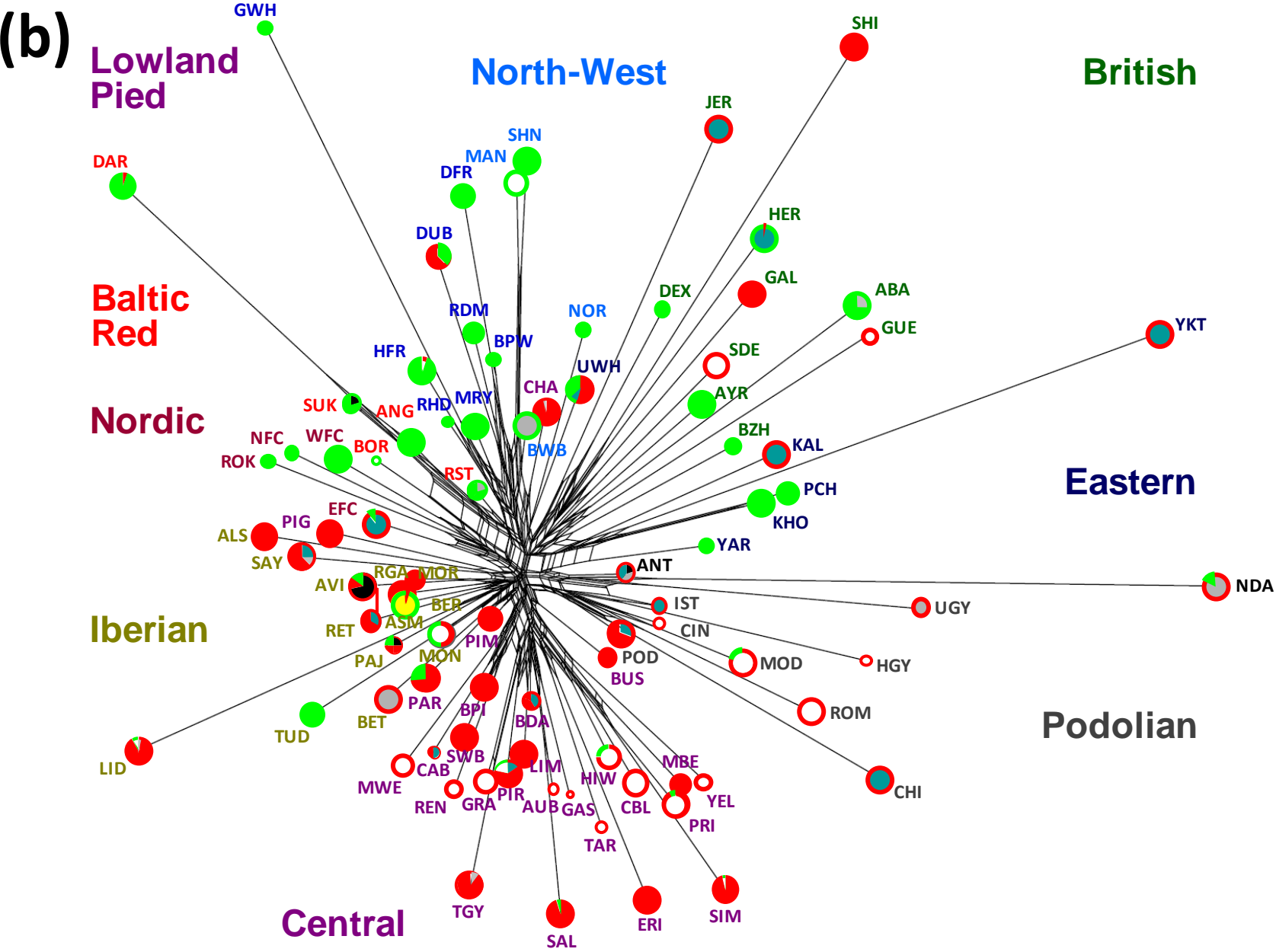

Supplement: Figure S2 — NeighborNet graphs of Reynolds' distances, based on 30 autosomal microsatellites. Haplotypes are indicated with the same colouring scheme as for: (a) maps in Figure S1, and (b) maps in Figure 1 . Abbreviations of breed names are given in Table 1. (PDF) [file pone.0015922.s002.pdf]
