# Supplementary material for: Dual Origins of Dairy Cattle Farming – Evidence from a Comprehensive Survey of European Y-Chromosomal Variation
Source: PLoS One. 2011 Jan 6;6(1):e15922. doi: 10.1371/journal.pone.0015922 (PMC3016991; doi:10.1371/journal.pone.0015922)
Supplement: Table S1 — (DOC) [file pone.0015922.s003.doc]

**Table S1. Number of samples genotyped for Y-chromosomal SNPs and the *ZFY* TG deletion.**

Several samples were analysed by more than one method, which revealed cosegregation of SNPs differentiating Y1 and Y2.

* The SNP typings do not differentiate between Y2 and Y3. Assignments to Y2 were based on the absence of Y3 in Europe (European samples only) and/or on an *INRA189* allele >90 bp, which has never been observed in zebu [22; unpublished results]. Assignments to Y3 were based on the *BM861* 156 bp allele, as well as the *INRA189* 88 bp allele, which both have so far never been observed in taurine cattle.

** Sequencing of the *ZFY*, *SRY* and *DBY* genes differentiated between the haplogroups at the following variable positions:

1. *ZFY* intron 10 to exon 11 [entries DQ336536 and DQ335637] – C614 in zebu; T614 in Y1 and Y2; T697-G698 deleted in Y1; G423 in Y1.
2. *ZFY* exon 11 [entries DQ336546 and DQ335647] – G71 in Y1; T71 in Y2 and Y3.
3. *SRY* [DQ336526 and DQ336527] – C1748, C2100 and T2372 in Y1 and Y2; T1748, T2100 and C2372 in Y3. Entry AY079139 [33] revealed three additional zebu-taurine differences (C771, G1329 and G1499).
4. *DBY* [DQ336556] – C385 in Y1 and Y2; T385 in Y3.
5. *DBY* [DQ336556 and DQ336557], composite microsatellite (343-358): (AT)^8^-(CT)^8^ in Y3; (AT)^10^-(CT)^8^ in most Y1 and Y2; sporadic other alleles in various breeds [in German Angus and Pirenaica (AT)^10^-(CT)^7^, in Belgian Blue, Asturiana de los Valles and Pirenaica (AT)^9^-(CT)^8^, in Pirenaica (AT)^11^-(CT)^7^ and (AT)^9^-(CT)^9^ almost fixed in Maremmana].

In addition, *UTY* [entry AY936543] has an additional polymorphism: G423 in Y1; T423 in Y2 and Y3 [14,22,25,31].

*** Only used for breeds not sampled in the present study.
