# Supplementary material for: Dual Origins of Dairy Cattle Farming – Evidence from a Comprehensive Survey of European Y-Chromosomal Variation
Source: PLoS One. 2011 Jan 6;6(1):e15922. doi: 10.1371/journal.pone.0015922 (PMC3016991; doi:10.1371/journal.pone.0015922)
Supplement: Table S2 — (DOC) [file pone.0015922.s004.doc]

**Table S2. Haplotype names used in this study.**

Compared with those published by Ginja *et al*. [22,30]; Kantanen *et al*. [31] and Pérez-Pardal *et al*. [14]. Bold printing indicates the two dominant haplotypes. *Doela and Telemark breeds were retyped as part of this study, and the *INRA189*-82 bp allele corresponds to *INRA189*-80 bp.
