# Supplementary material for: Dual Origins of Dairy Cattle Farming – Evidence from a Comprehensive Survey of European Y-Chromosomal Variation
Source: PLoS One. 2011 Jan 6;6(1):e15922. doi: 10.1371/journal.pone.0015922 (PMC3016991; doi:10.1371/journal.pone.0015922)
Supplement: Table S3 — (DOC) [file pone.0015922.s005.doc]

**Table S3. AMOVA results and F statistics for the 12 geographic groups, including all 138 breeds.**
