# Supplementary material for: Dual Origins of Dairy Cattle Farming – Evidence from a Comprehensive Survey of European Y-Chromosomal Variation
Source: PLoS One. 2011 Jan 6;6(1):e15922. doi: 10.1371/journal.pone.0015922 (PMC3016991; doi:10.1371/journal.pone.0015922)
Supplement: Table S4 — (DOC) [file pone.0015922.s006.doc]

**Table S4. F*st* values obtained from haplotype frequencies.**

Regions with relatively low F*st* values are highlighted in yellow. Results significant at the 5% level, but not high, are shown in orange. The lowest F*st* value, and not significantly different from 0, is highlighted in red and bold.
